# The Validity of a New Low-Dose Stereoradiography System to Perform 2D and 3D Knee Prosthetic Alignment Measurements

**DOI:** 10.1371/journal.pone.0146187

**Published:** 2016-01-15

**Authors:** Marrigje F. Meijer, Ton Velleman, Alexander L. Boerboom, Sjoerd K. Bulstra, Egbert Otten, Martin Stevens, Inge H. F. Reininga

**Affiliations:** 1 Department of Orthopedics, University of Groningen, University Medical Center Groningen, Groningen, The Netherlands; 2 Department of Radiology, University of Groningen, University Medical Center Groningen, Groningen, The Netherlands; 3 Center for Human Movement Sciences, University of Groningen, University Medical Center Groningen, Groningen, The Netherlands; 4 Department of Trauma Surgery, University of Groningen, University Medical Center Groningen, Groningen, The Netherlands; Rutgers University -New Jersey Medical School, UNITED STATES

## Abstract

**Introduction:**

The EOS stereoradiography system has shown to provide reliable varus/valgus (VV) measurements of the lower limb in 2D (VV2D) and 3D (VV3D) after total knee arthroplasty (TKA). Validity of these measurements has not been investigated yet, therefore the purpose of this study was to determine validity of EOS VV2D and VV3D.

**Methods:**

EOS images were made of a lower limb phantom containing a knee prosthesis, while varying VV angle from 15° varus to 15° valgus and flexion angle from 0° to 20°, and changing rotation from 20° internal to 20° external rotation. Differences between the actual VV position of the lower limb phantom and its position as measured on EOS 2D and 3D images were investigated.

**Results:**

Rotation, flexion or VV angle alone had no major impact on VV2D or VV3D. Combination of VV angle and rotation with full extension did not show major differences in VV2D measurements either. Combination of flexion and rotation with a neutral VV angle showed variation of up to 7.4° for VV2D; maximum variation for VV3D was only 1.5°. A combination of the three variables showed an even greater distortion of VV2D, while VV3D stayed relatively constant. Maximum measurement difference between preset VV angle and VV2D was 9.8°, while the difference with VV3D was only 1.9°. The largest differences between the preset VV angle and VV2D were found when installing the leg in extreme angles, for example 15° valgus, 20° flexion and 20° internal rotation.

**Conclusions:**

After TKA, EOS VV3D were more valid than VV2D, indicating that 3D measurements compensate for malpositioning during acquisition. Caution is warranted when measuring VV angle on a conventional radiograph of a knee with a flexion contracture, varus or valgus angle and/or rotation of the knee joint during acquisition.

## Introduction

Achieving optimal prosthetic alignment during total knee arthroplasty (TKA) is crucial, as malalignment is associated with worse functional outcome, earlier aseptic loosening of the prosthesis and, eventually, revision surgery (rTKA) [1–6].

Long leg standing radiographs (LLRs) are frequently used to measure knee prosthesis alignment in the coronal plane. Assessment of alignment with LLRs in the sagittal plane is not common practice, and mostly lateral radiographs of the knee are used. With LLR this 2D measurement technique has some pitfalls though. Due to divergence in the horizontal and vertical planes, measured angles may not be correct. More importantly, validity of the measurements is easily influenced by the position of the patient’s lower limb during acquisition. Varus or valgus angle of the knee, rotation and flexion of the lower limb are shown to influence alignment measurements [7–10].

To tackle this issue, 3D measurements such as CT-scanning have been developed to perform these measurements, but major drawbacks of this technique are the costs and high doses of radiation. Furthermore, CT-scanning is done in non-weight-bearing position. The EOS system (EOS Imaging, Paris, France) [11] is a new alternative: its 3D measurements of this system are based on two orthogonally made LLRs done on a 1:1 scale. This system uses an even lower dose of radiation than normal X-rays [11,12]. Since the leg is scanned by a C-arm that moves up and down while the patient is standing, divergence in the vertical plane is diminished and images are weight-bearing.

The EOS system uses sterEOS software (EOS Imaging, Paris, France) to create EOS 3D reconstructions of the LLRs made. This software is originally designed for lower limbs not containing a knee prosthesis. We have developed a protocol to perform 3D varus/valgus (VV3D) measurements of the lower limb on patients with a knee prosthesis, and concluded that this measurement protocol has excellent intra- and interobserver reliability [13]. In this study, significant differences between EOS 2D varus/valgus (VV2D) and VV3D measurements were found. It was hypothesized that the 2D measurement might be influenced by malpositioning during acquisition while the 3D measurement mathematically corrects for this issue, but validity of this 3D measurement protocol for lower limbs containing a knee prosthesis has not been investigated yet.

Hence the aim of this study was to investigate the validity of EOS VV2D and VV3D measurements in a lower limb containing a knee prosthesis. As no gold standard for performing knee prosthesis alignment measurements exists, we designed a phantom study using a lower limb phantom containing a knee prosthesis in which we could alter varus/valgus (VV), flexion and rotation of the lower limb while obtaining EOS images.

## Materials and Methods

**Fig 1:**
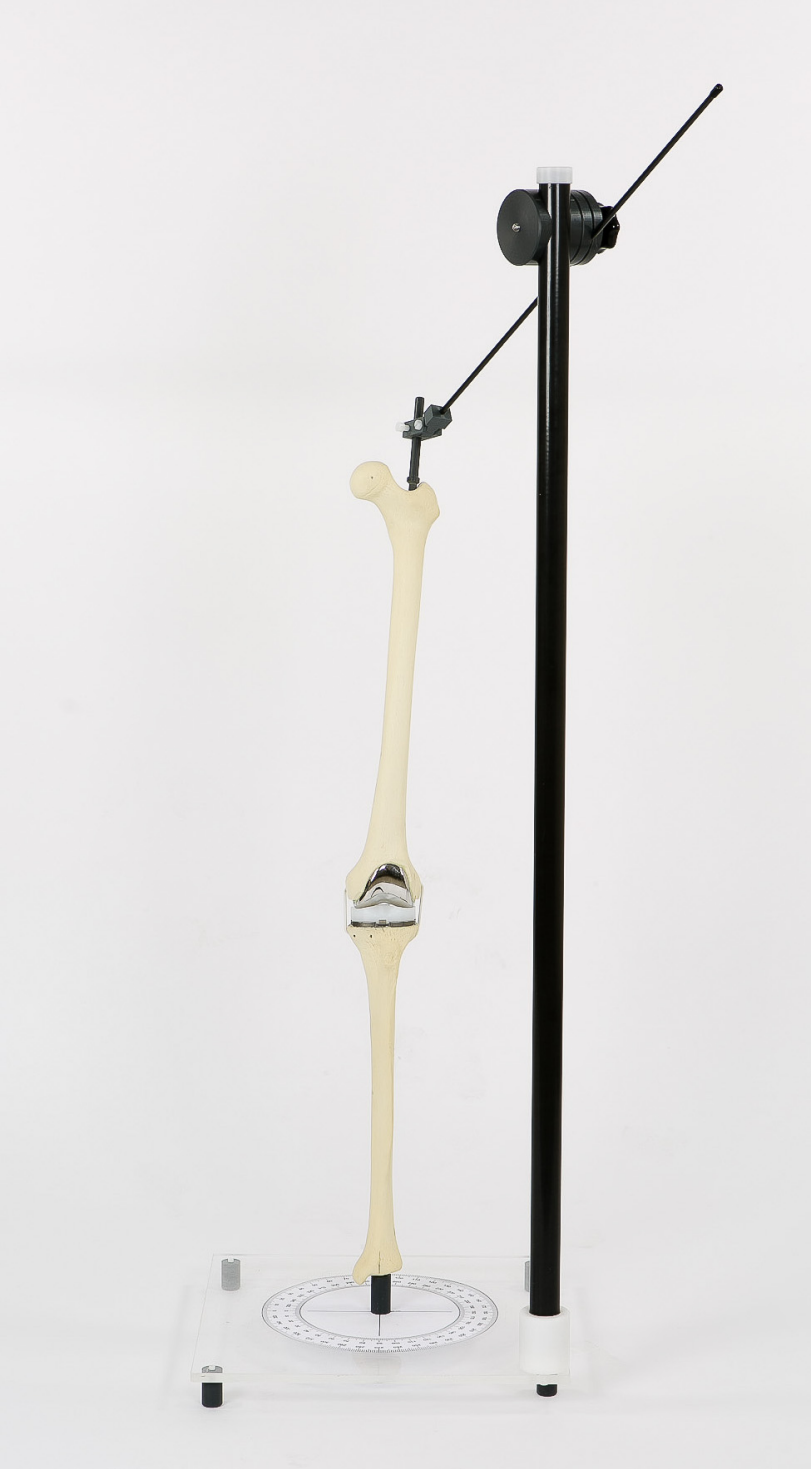
The experimental setup.

A lower limb phantom (Sawbones^®^ Inc., Vashon Island, WA, USA) containing a NexGen Legacy Posterior Stabilized Knee^®^ prosthesis was used. The lower limb was fixed into a frame in which it was possible to change VV and flexion/extension angle of the knee by moving the femur. The distal tibia was fixed to the base plate (Fig 1). An extendable goniometer (Lafayette Gollehon extendable goniometer, model 01135) was used to set the lower limb in different varus/valgus and flexion/extension positions. The protractor at the base of the construction was used to place the construction in different angles of rotation during acquisition.

VV was varied from 15° varus to 15° valgus with 5° increments. A negative value (-) indicated varus and a positive value (+) indicated valgus. Flexion was varied from 0° to 20° with 5° increments. Rotation was varied from 20° internal rotation to 20° external rotation. A negative value (-) indicated internal rotation and a positive value (+) indicated external rotation. The influence of these three variations in lower-limb position on EOS VV2D and VV3D measurements were investigated both separately and combined. Settings of the EOS system during acquisition of the images was scan speed 2 (6 in/s), voltage 55 kV and amperage 32 mA.

All EOS VV2D and VV3D measurements were performed by a radiology assistant (TV) who had extensive experience in taking such measurements and who was blinded to the preset lower limb-positions. SterEOS software (EOS Imaging, Paris, France) was used to perform VV2D measurements on the anterior-posterior (AP) image and VV3D measurements on the AP and lateral (LAT) images. The measurement protocol that was used is described extensively in our previous publication [13].

**Fig 2:**
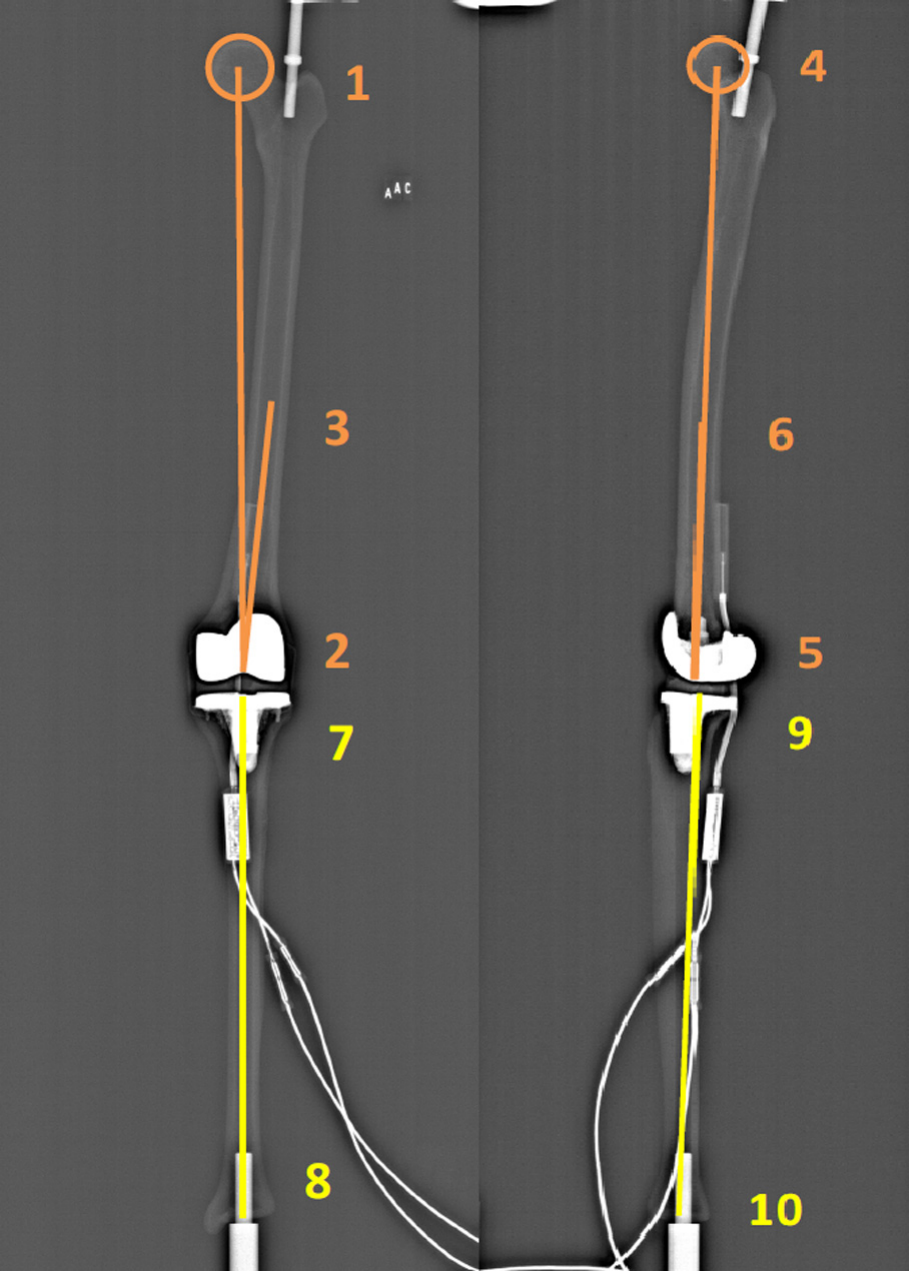
Identification of the landmarks on the frontal and lateral images.

In order to calculate coronal and sagittal alignment parameters of the lower limb in 2D and 3D, the “lower limb alignment” mode was used. The first step was to define the left or right lower limb and to choose the modeling “lower limb alignment” mode. Next, identification of the lower limb on the AP and LAT images was done in 10 steps (Fig 2):

Femur:

- center of the femoral head (points 1 and 4);
- center of the distal femoral notch (points 2 and 5);
- center of the diaphysis in its distal third (points 3 and 6).

Tibia:

- center of the tibial plateau. the axis from the center of the ankle to the center of the tibial plateau represents the anatomical axis of the tibia (points 7 and 9);
- center of the distal articular surface in the upper ankle joint (points 8 and 10).

**Fig 3:**
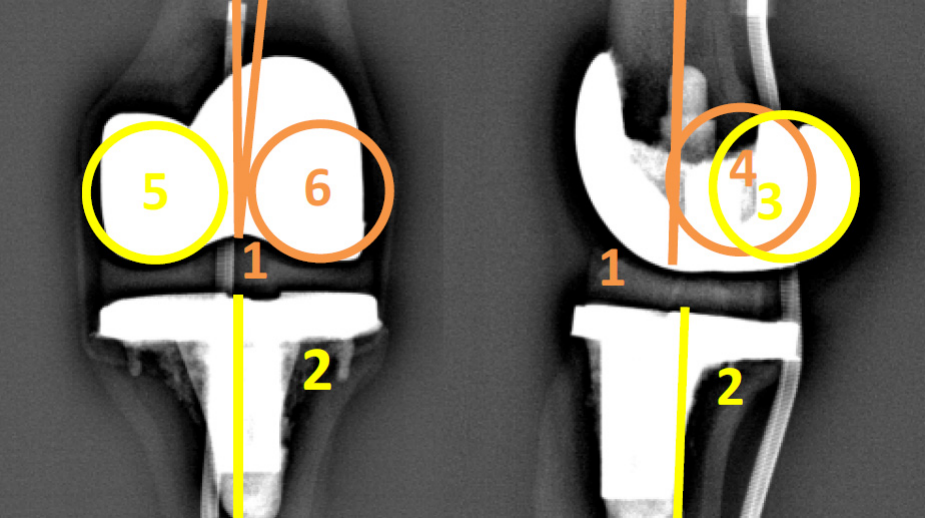
Adjustment of the landmarks on the frontal and lateral images.

The next step was adjustment of the landmarks in four steps (Fig 3):

1. Adjustment of the position of the sphere of the femoral head in both views. It is possible to enlarge or minimize the size of the sphere according to the size and shape of the femoral head, in order to mark the center of the femoral head as precisely as possible;
2. Adjustment of the point in the center of the distal third of the femoral diaphysis;
3. Adjustment of the position of the point in the center of the femoral notch and tibial plateau, and marking of the femoral condyles. The condyles have to be identified on the AP and LAT images using the two spheres. It is possible to adjust the size of the spheres according to the size of the condyles. On the AP image the center of the spheres has to be located in the center of each condyle. On the LAT image the spheres have to be tangent to the posterior part of the condyles. It is important not to confuse the medial with the lateral condyles. In order to identify the right condyle, the epipolar line is used to differentiate between the two condyles by observing the correspondence of condylar height on both the AP and the LAT image;
4. Adjustment of the reference point in the center of the distal articular surface on the AP and LAT images.

VV2D is the angle between the mechanical axis of the femur (axis between points 1 and 2) and the tibia (axis between points 7 and 8) on the AP image (Fig 2). For the 3D measurement, the points marked on the AP and LAT (Fig 2) images as described above are combined to generate the mechanical axes of the femur and tibia. VV3D is the angle between the three-dimensional mechanical axis of the femur (axis between points 1–4 and 2–5) and tibia (axis between point 7–9 and 8–10).

### Statistical analyses

All statistical analyses were performed using the IBM SPSS Statistics for Windows software (Version 22.0, IBM Corp., Armonk, NY, USA). Intraobserver reliability of setup installation was investigated. To that end, seven different combinations of VV, flexion and rotation were installed twice. VV angle of the first setup was compared to the second setup for both 2D and 3D. Relative intraobserver reliability of the setup was investigated by calculating the Intraclass Correlation Coefficients (ICCs) [14]. ICCs were interpreted according to the benchmarks described by Fleiss [15]: an ICC >0.75 represents an excellent correlation, 0.40–0.75 moderate-to-good and <0.40 represents a poor correlation [15]. The Bland & Altman method was used to investigate absolute intraobserver reliability of the setup [16]. Mean difference and 95% confidence interval (CI) between measurements 1 and 2 were calculated. When zero lies in the 95% CI, no systematic bias exists between the measurements [16].

To compare the VV2D and VV3D for different positions of the lower limb phantom, the mean absolute differences and range of the absolute differences were used. Mean difference was not calculated, as varus and valgus are negative and positive values respectively, thus calculating the mean leads to an underestimation of the differences. On the other hand, the range of the absolute differences might give an underestimation of the effect, due to elimination of varus or valgus direction. For this reason, besides the range of the absolute differences we also showed the original range.

## Results

Relative intraobserver reliability for setup installation was excellent, with an ICC of 0.98 (95% CI: 0.94–1.00) for both VV2D and VV3D. Absolute intraobserver reliability did not show a systematic bias for either VV2D (95% CI: -1.51–0.15) (S1 Fig) or VV3D (95% CI: -2.12–0.02) (S2 Fig).

**Fig 4:**
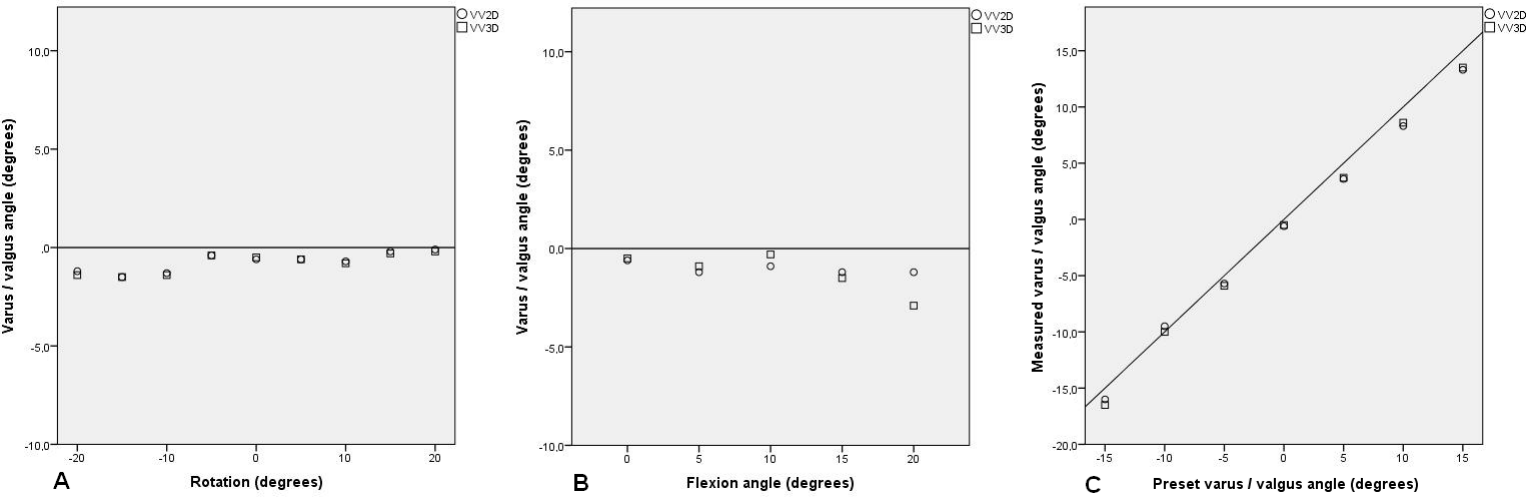
The influence of rotation, flexion and varus/valgus angle on EOS 2D and 3D varus/valgus measurements and measurement errors.

The results of the influence of the preset VV and flexion angles alone and combined on the VV2D and VV3D are shown in Table 1. When the leg was positioned in 0° VV and 0° flexion and the construction was rotated from -20° to 20° with 5° increments, the mean absolute difference between the preset VV angle and VV2D was 0.73° (SD: 0.49; range: 0.1°–1.5°) and the mean absolute difference between the preset VV angle and VV3D was 0.79° (SD: 0.51; range: 0.2°–1.5°) (Table 1 and Fig 4A). When the leg was positioned in 0° VV and 0° rotation while varying the flexion angle from 0° to 20° with 5° increments, the mean absolute difference between the preset VV angle and VV2D was 1.02° (SD: 0.27; range: 0.6°–1.2°) and the mean absolute difference between the preset VV angle and VV3D was 1.22° (SD: 1.04; range: 0.3°–2.9°) (Fig 4B). When the leg was positioned in 0° flexion and rotation while varying the VV from 15° varus to 15° valgus with 5° increments, the mean absolute difference between the preset VV angle and the VV2D was 1.09° (SD: 0.51; range 0.5°–1.7°) and the mean absolute difference between the preset VV angle and VV3D was 1.01° (SD: 0.58; range: 0.0°–1.5°) (Fig 4C).

**Table 1:** Results of the measured varus/valgus angles in 2D and 3D for different positions.

Varus/valgus	Flexion	Mean abs diff 2D (SD)	Range abs diff 2D	Range diff 2D	Mean abs diff 3D (SD)	Range abs diff 3D	Range diff 3D
0	0	0.73 (0.49)	0.1–1.5	0.1–1.5	0.79(0.51)	0.2–1.5	0.2–1.5
5	0	1.62 (0.15)	1.4–1.8	1.4–1.8	1.38(0.28)	1.0–1.7	1.0–1.7
-5	0	0.62 (0.13)	0.4–0.7	0.4–0.7	1.14(0.63)	0.7–2.2	0.7–2.2
10	0	1.82 (0.26)	1.6–2.1	1.6–2.1	1.28(0.65)	0.3–1.9	0.3–0.9
-10	0	0.72 (0.57)	0.3–1.7	-1.7 –-0.3	0.46(0.30)	0.0–0.7	-0.7–0.7
15	0	2.14 (0.49)	1.7–2.9	1.7–2.9	1.40(0.54)	0.7–1.9	0.7–1.9
-15	0	0.64 (0.39)	0.1–1.0	-0.1–1.0	1.56(0.49)	1.0–2.2	1.0–2.2
0	10	2.2 (1.38)	0.9–4.2	-2.3–4.2	0.5(0.25)	0.3–0.9	0.3–0.9
0	20	4.54 (2.88)	1.2–8.6	-5.2–8.6	2.42(0.65)	1.8–3.3	1.8–3.3
5	10	2.04 (1.46)	0.5–4.1	-1.9–4.1	0.60(0.33)	0.2–0.9	-0.2–0.9
5	20	3.92 (2.23)	1.4–6.8	-6.8–5.3	0.96(1.15)	0.3–3.0	-3.0–0.4
10	10	2.2 (1.43)	0.1–4.0	-2.9–4.0	0.32(0.29)	0.1–0.8	-0.1–0.8
10	20	4.52 (2.61)	1.7–8.2	-8.2–5.6	1.06(0.85)	0.0–2.3	-2.3–0
15	10	2.62 (1.22)	1.3–4.2	-3.2–4.2	0.50(0.34)	0.0–0.9	-0.9–0.7
15	20	5.6 (3.59)	1.3–10.5	-10.5–6	1.16(0.55)	0.2–1.6	-1.3–1.6
-5	10	2.24 (1.61)	0.2–4.4	-1.8–4.4	0.84(0.21)	0.6–1.1	0.6–1.1
-5	20	4.00 (2.43)	0.9–7.1	-5.3–7.1	0.52(0.49)	0.0–1.3	0.0–1.3
-10	10	2.42 (1.84)	0.0–4.9	-2.0–4.9	2.84(0.78)	1.8–3.8	1.8–3.8
-10	20	3.68 (2.24)	0.6–6.2	-6.2–5.5	2.06(0.38)	1.5–2.5	-2.5 –-1.5
-15	10	2.50 (1.63)	0.7–4.9	-4.9–3.1	0.46(0.26)	0.2–0.8	-0.8 –-0.2
-15	20	5.00 (3.72)	0.8–10.6	-4.4–10.6	2.18(0.72)	1.1–3.0	1.1–3.0

The synthetic leg was positioned in a preset varus/valgus and flexion angle of the knee, while rotation of the construction was varied from 20° internal rotation to 20° external rotation with 5° increments. Angles are expressed in degrees (°).Abbreviations: Mean abs diff 2D = mean absolute difference between preset varus/valgus angle and varus/valgus angle measured in 2D; SD = standard deviation; Range abs diff 2D = range of absolute difference between preset varus/valgus angle and varus/valgus angle measured in 2D; Range diff 2D = range of difference between preset varus/valgus angle and varus/valgus angle measured in 2D; Mean abs diff 3D = mean absolute difference between preset varus/valgus angle and varus/valgus angle measured in 3D; Range abs diff 3D = range of absolute difference between preset varus/valgus angle and varus/valgus angle measured in 3D; Range diff 3D = range of difference between preset varus/valgus angle and varus/valgus angle measured in 3D.

**Fig 5:**
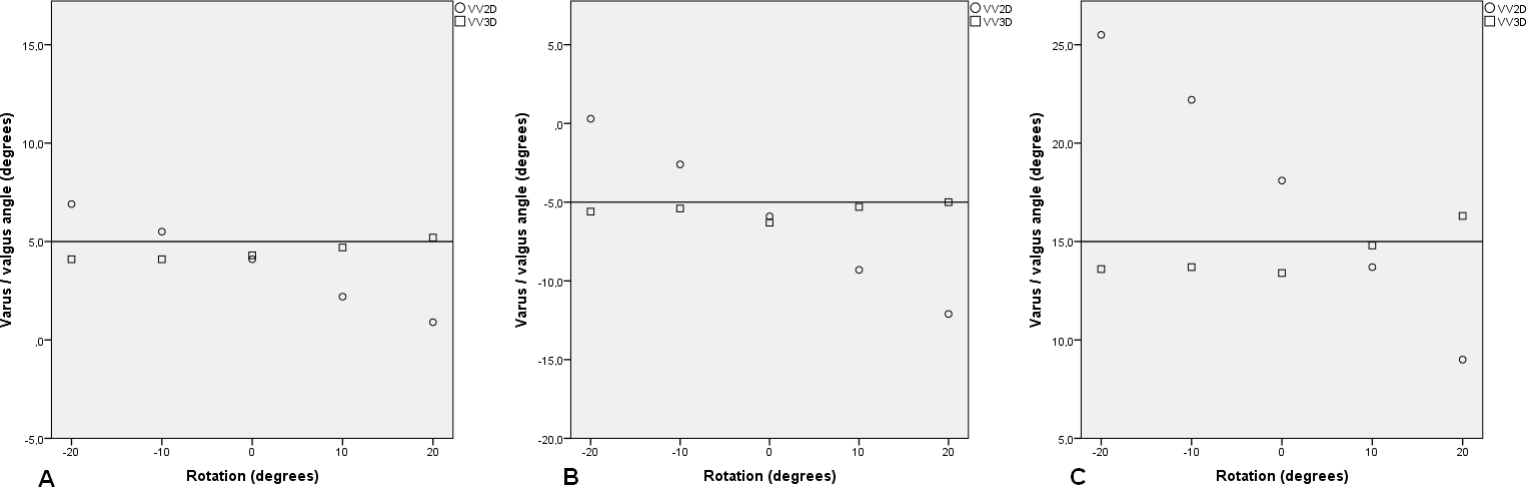
EOS 2D/3D measurements and measurement errors of varus/valgus angle for different combinations of preset varus/valgus and flexion angle and rotation.

Three different combinations of VV and flexion angles are shown in Fig 5. In Fig 5A the preset angles were 5° valgus and 10° flexion. In Fig 5B the preset angles were 5° varus and 20° flexion. In Fig 5C the preset angles were 15° valgus and 20° flexion.Scatter dots of other combinations of VV and flexion angles are added in S1 Appendix.

## Discussion

With the EOS system it is possible to measure knee prosthesis alignment measurements in 2D and 3D. Intra- and interobserver reliability and validity for measuring VV angle in 2D and 3D of lower limbs not containing a knee prosthesis are shown to be excellent [17,18]. Intra- and interobserver reliability for measuring VV angle in 2D and 3D after TKA are also shown to be excellent [13], but validity has not been investigated yet. Previous research demonstrated that significant differences exist between EOS VV2D and VV3D measurements [13]. It was hypothesized that VV angle and malpositioning during acquisition influenced 2D measurements, but not 3D measurements. Aim of this study was therefore to study the validity of EOS 2D and 3D VV measurements using a lower limb phantom containing a knee prosthesis.

Our results showed that validity of VV3D is good, but VV2D showed considerable variation. Rotation, flexion or VV angle alone did not have a major impact on VV2D or VV3D. The combination of VV angle and rotation in full extension did not show major differences in VV2D or VV3D measurements either. The combination of flexion and rotation with a neutral VV angle showed variation of up to 13.8° for VV2D, while maximum variation for VV3D was only 1.5°. A combination of the three variables demonstrated an even greater variety. Maximum measurement difference of VV2D was 16.5°, while VV3D differed only 2.9° in that same setup. The largest differences between the preset VV angle and the VV2D measurement error were found with the leg in extreme positions, for example 15° valgus, 20° flexion and 20° internal rotation.

To our knowledge, no studies have been conducted in which a lower limb phantom containing a knee prosthesis was used to investigate the influence of VV angle and/or malpositioning on VV measurements. There are however studies in which a lower limb phantom containing no prosthetic material was used. Radtke et al. [9] reported on the influence of rotation (-20°–20°) on the VV measurements on conventional AP radiographs. The lower limb phantom used was set at 6.5° valgus. The measured VV angle varied from 4.6° to 6.8° valgus. This is in line with our conclusion that a VV angle in combination with rotation does not cause large variations in VV2D. Lonner et al. [7] found that the combination of 5° valgus, 10° flexion and rotation varying from 25° internal to 20° external rotation caused a significant difference of 4.4° between preset VV angle and VV2D. We used a similar setup but a maximum internal rotation of 20° and found a difference of 6.0° between preset VV angle and VV2D, which is comparable to the results of Lonner et al. [7]. Swanson et al. [10] concluded that VV2D is more sensitive to rotation in combination with VV angle; in their study, the anatomical axis of the lower limb phantom was set at 18° valgus and 0° flexion. When rotating from 10° internal rotation to 20° external rotation the measured anatomical angle in 2D ranged from 20.2° to 13.6° valgus respectively. In our study the effect was not as outspoken. When we set the lower limb phantom at a mechanical axis of 15° valgus and 0° flexion, the maximum difference between the preset VV angle and the VV2D was 2.9°; at a mechanical axis of 15° varus and 0° flexion the maximum difference between the preset VV angle and the VV2D was 1.0°. Brouwer et al. [8] investigated the influence of flexion and rotation alone and in combination on the VV angle measured on conventional X-rays. The cadaveric leg used in their study had a VV angle of 10° varus. They found that flexion of the knee (from 0° to 30°) or rotation of the lower limb (from -30°–30°) separately had very little effect on the angles measured on the AP radiograph. Simultaneous flexion and rotation, however, caused large changes (up to 28°) in the measured VV angle. This is in line with the results of our study. A combination of VV angle with flexion alone did not show great variety in the VV2D in our study either, but when rotation was added VV2D varied significantly.

Differences between VV2D and VV3D EOS measurements have been reported previously. Thelen et al. [19] conducted a phantom study with a lower limb phantom without a knee prosthesis to investigate the influence of flexion and rotation in VV2D and VV3D. The preset VV angle of the lower limb was 5° valgus. They found that the combination of 5° valgus, flexion (up to 18°) and rotation provoked VV2D measurement errors up to 6.8°, compared to 1.5° for VV3D. Their conclusion was that 3D modeling allows for more valid evaluation of coronal alignment than 2D, eliminating bias due to an abnormal knee positioning. The original SterEOS measurement protocol, as also used in the study of Thelen et al. [19], is designed for lower limbs not containing a knee prosthesis. Since no official measurement protocol existed for performing these measurements after TKA, we drew up a measurement protocol [13], and validity of that protocol is investigated in this study. We also wanted to evaluate the influence of VV angle on VV2D and VV3D further, and varied this angle from 15° varus to 15° valgus. No previous studies with or without knee prosthesis *in situ* have investigated the influence of 15° varus or valgus on the effect on VV2D measurements. Thelen et al. [19] fixed their phantom at 5° valgus. We added the extreme positions of 15° varus to 15° valgus to the analyses, as these deformities do occur in clinical practice. Results of our study are comparable to those of Thelen et al. [19]: we also concluded that VV3D measurements are more valid than VV2D measurements, since VV3D corrects for malpositioning during acquisition while VV2D does not.

This study has some limitations. First of all, the study was conducted with use of a lower limb phantom, therefore the EOS images differed from those obtained from patients. Still, with adjustment of the scan speed, voltage and amperage settings we were able to get good visualization of the bony structures. Secondly, it was not possible to compare validity of the EOS system to a gold standard, as there isn’t one for measuring VV angle. We therefore designed this experimental setup using lower limb phantom and an extendable goniometer. Despite not having a gold standard, installing the lower limb phantom showed excellent intraobserver reliability, the influence of malpositioning and angle on 2D measurements was clear, and 3D measurements stayed relatively constant.

To our knowledge, this is the first study to investigate validity of EOS VV2D and VV3D measurements in a leg containing a knee prosthesis. Our study showed that EOS VV3D measurements are more valid than EOS VV2D measurements, since VV2D measurements are influenced by VV angle and malpositioning. A combination of flexion and rotation caused major variation in VV2D measurements. A combination of varus or valgus angle, flexion and rotation caused an even larger variation in VV2D measurements.

In clinical practice a combination of flexion deformity and VV deformity of the knee frequently meet, and one should pay extra attention when positioning the patient so as to obtain an LLR without adding a rotation error. Orthopedic surgeons should also be aware of this phenomenon, and caution is warranted when measuring VV angle on a conventional radiograph when the patient has a knee with a flexion contracture, varus or valgus angle of the knee and/or is standing with a rotated knee joint during acquisition. Hence it can be concluded that EOS 3D reconstructions are a valid and reliable method for measuring varus/valgus angle of the lower limb after TKA. EOS 3D reconstructions are superior to conventional anteroposterior LLRs, as EOS 3D measurements will be corrected for unseen deformities and malpositioning.

## Supporting Information

S1 Appendix The influence of rotation, flexion and varus/valgus angle on EOS 2D and 3D varus/valgus measurements. (DOCX)

S1 Fig Absolute intraobserver reliability of the setup for VV2D. (TIF)

S2 Fig Absolute intraobserver reliability of the setup for VV3D. (TIF)
