# Supplementary material for: The Validity of a New Low-Dose Stereoradiography System to Perform 2D and 3D Knee Prosthetic Alignment Measurements
Source: PLoS One. 2016 Jan 15;11(1):e0146187. doi: 10.1371/journal.pone.0146187 (PMC4714906; doi:10.1371/journal.pone.0146187)
Supplement: S1 Appendix — (DOCX) [file pone.0146187.s001.docx]

**The influence of rotation, flexion and varus/valgus angle on EOS 2D and 3D varus/valgus measurements**


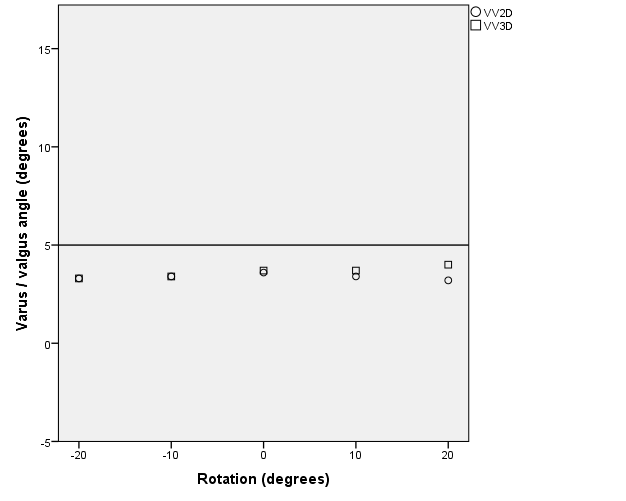


Fig 1. The preset varus/valgus was 5° valgus, flexion angle was 0° and rotation was varied from 20° internal rotation to 20° external rotation with 5° increments.


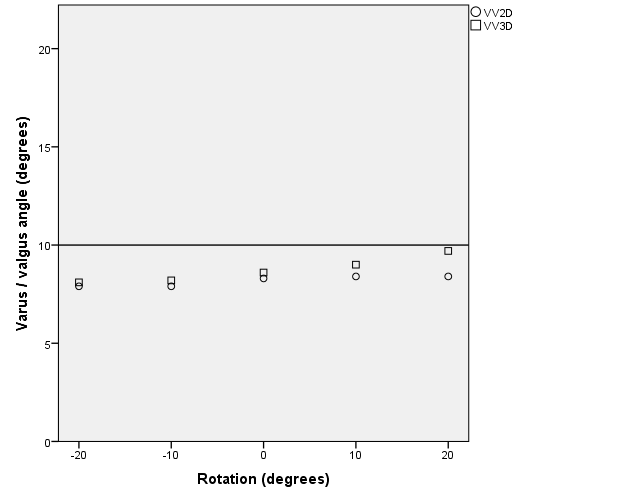


Fig 2. The preset varus/valgus was 10° valgus, flexion angle was 0° and rotation was varied from 20° internal rotation to 20° external rotation with 5° increments.


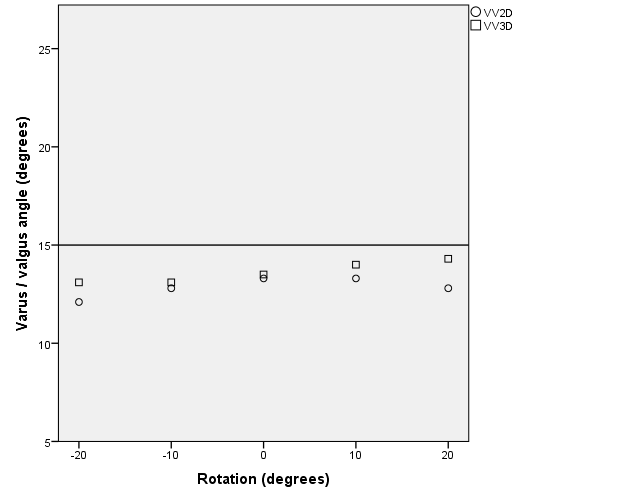


Fig 3. The preset varus/valgus was 15° valgus, flexion angle was 0° and rotation was varied from 20° internal rotation to 20° external rotation with 5° increments.


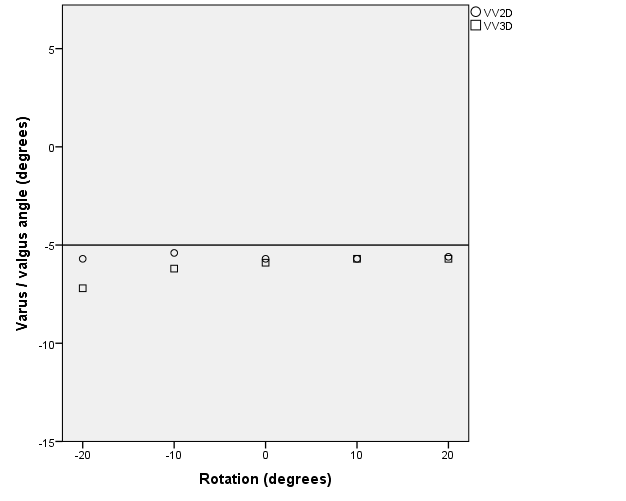


Fig 4. The preset varus/valgus was 5° varus, flexion angle was 0° and rotation was varied from 20° internal rotation to 20° external rotation with 5° increments.


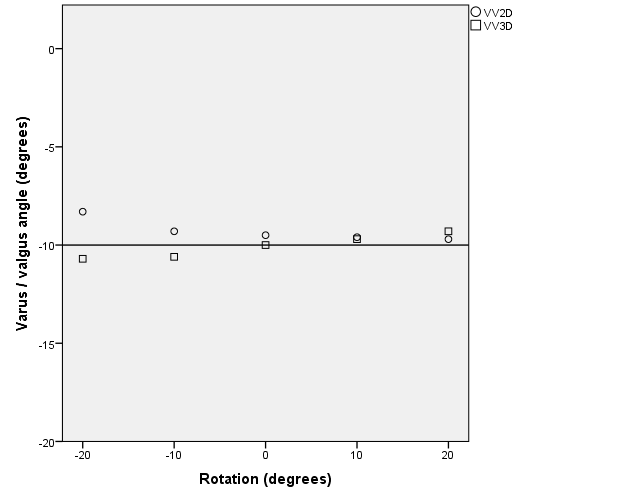


Fig 5. The preset varus/valgus was 10° varus, flexion angle was 0° and rotation was varied from 20° internal rotation to 20° external rotation with 5° increments.


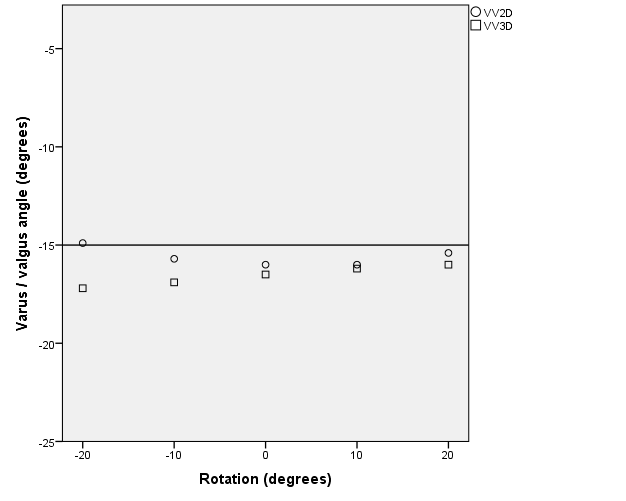


Fig 6. The preset varus/valgus was 15° varus, flexion angle was 0° and rotation was varied from 20° internal rotation to 20° external rotation with 5° increments.


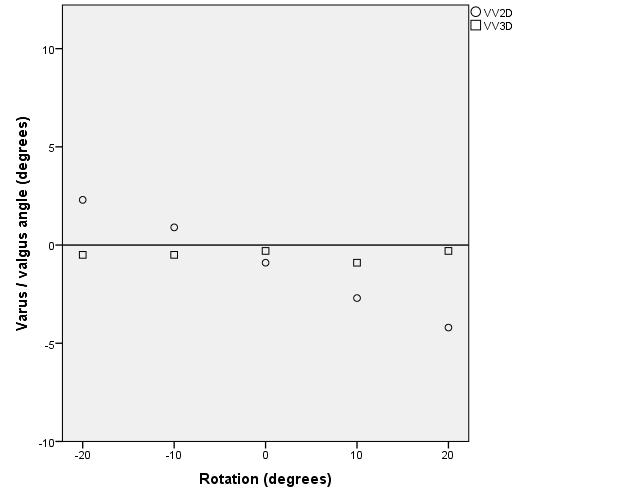


Fig 7. The preset varus/valgus was 0°, flexion angle was 10° and rotation was varied from 20° internal rotation to 20° external rotation with 5° increments.


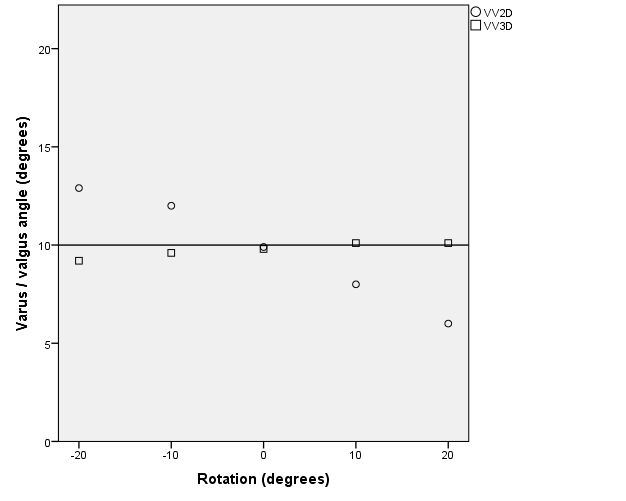


Fig 8. The preset varus/valgus was 10° valgus, flexion angle was 10° and rotation was varied from 20° internal rotation to 20° external rotation with 5° increments.


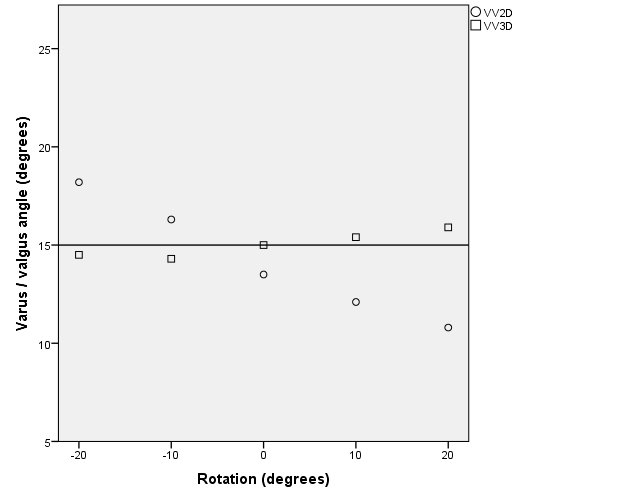


Fig 9. The preset varus/valgus was 15° valgus, flexion angle was 10° and rotation was varied from 20° internal rotation to 20° external rotation with 5° increments.


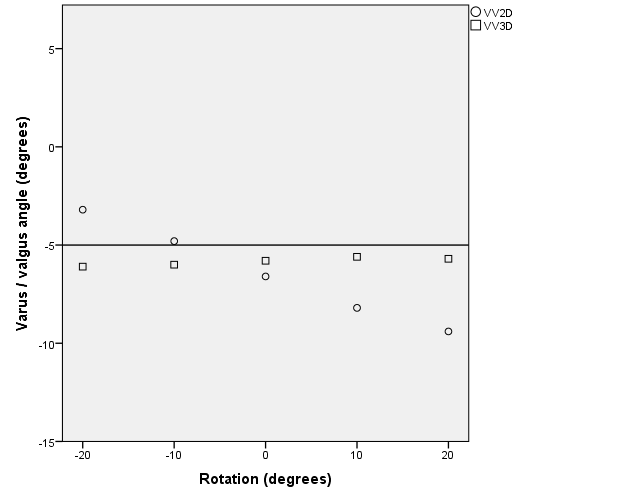


Fig 10. The preset varus/valgus was 5° varus, flexion angle was 10° and rotation was varied from 20° internal rotation to 20° external rotation with 5° increments.


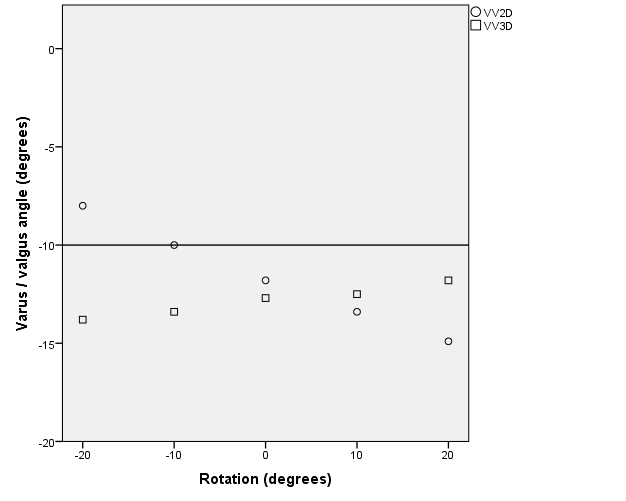


Fig 11. The preset varus/valgus was 10° varus, flexion angle was 10° and rotation was varied from 20° internal rotation to 20° external rotation with 5° increments.


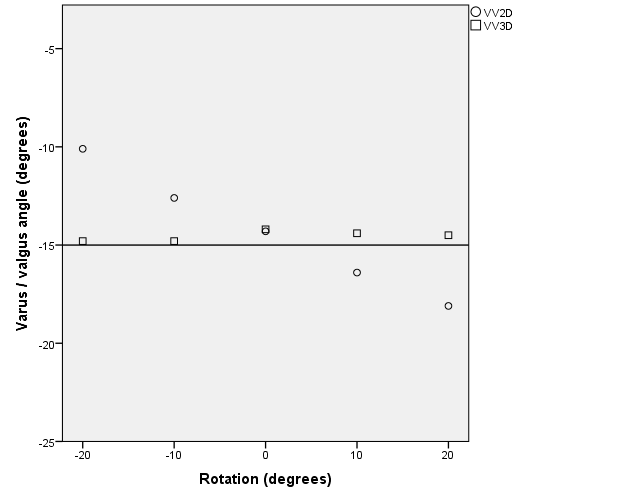


Fig 12. The preset varus/valgus was 15° varus, flexion angle was 10° and rotation was varied from 20° internal rotation to 20° external rotation with 5° increments.


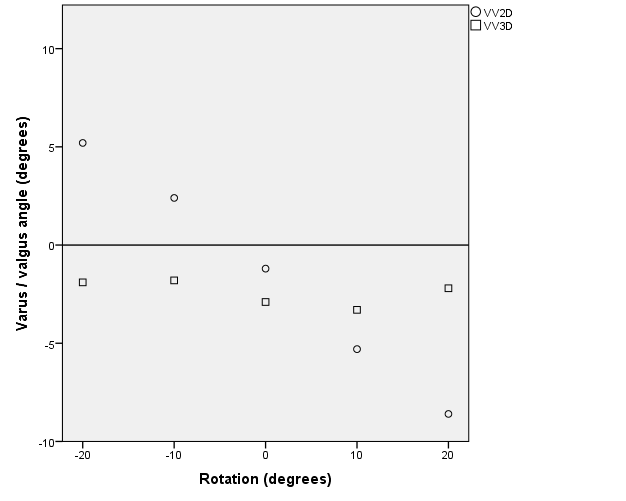


Fig 13. The preset varus/valgus was 0°, flexion angle was 20° and rotation was varied from 20° internal rotation to 20° external rotation with 5° increments.


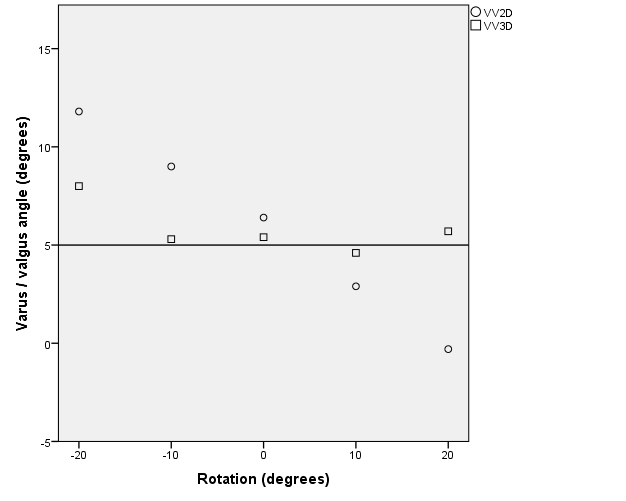


Fig 14. The preset varus/valgus was 5° valgus, flexion angle was 20° and rotation was varied from 20° internal rotation to 20° external rotation with 5° increments.


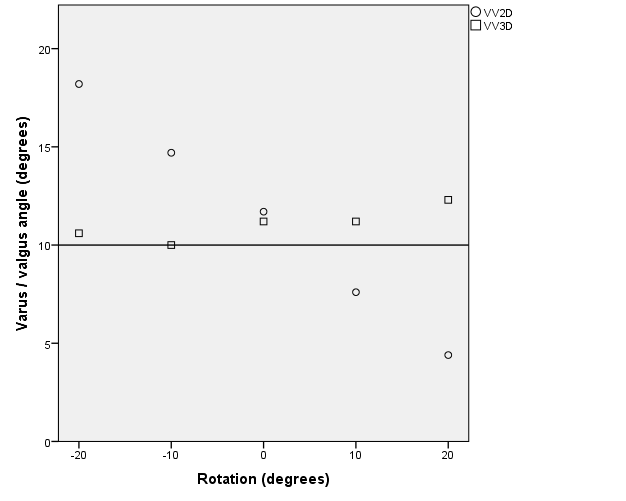


Fig 15. The preset varus/valgus was 10° valgus, flexion angle was 20° and rotation was varied from 20° internal rotation to 20° external rotation with 5° increments.


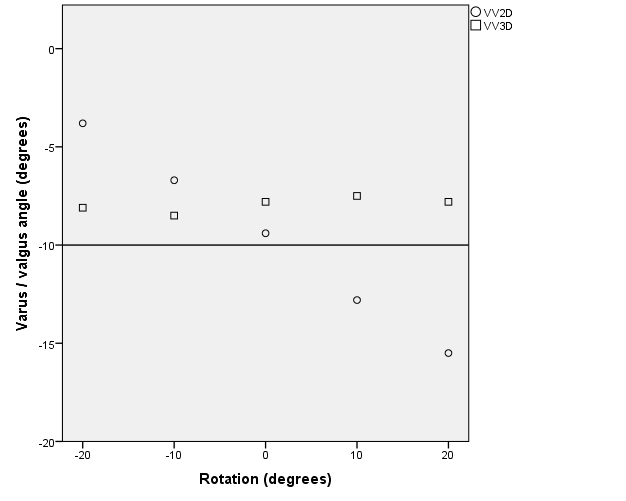


Fig 16. The preset varus/valgus was 10° varus, flexion angle was 20° and rotation was varied from 20° internal rotation to 20° external rotation with 5° increments.


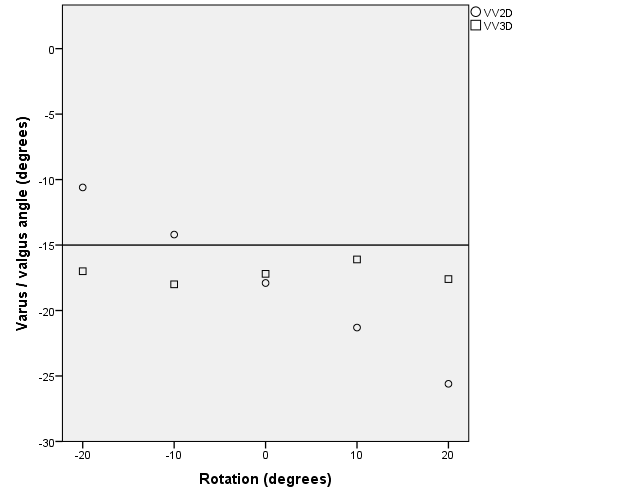


Fig 17. The preset varus/valgus was 15° varus, flexion angle was 20° and rotation was varied from 20° internal rotation to 20° external rotation with 5° increments.
