# Supplementary figures and images for: The Validity of a New Low-Dose Stereoradiography System to Perform 2D and 3D Knee Prosthetic Alignment Measurements
Source: PLoS One. 2016 Jan 15;11(1):e0146187. doi: 10.1371/journal.pone.0146187 (PMC4714906; doi:10.1371/journal.pone.0146187)

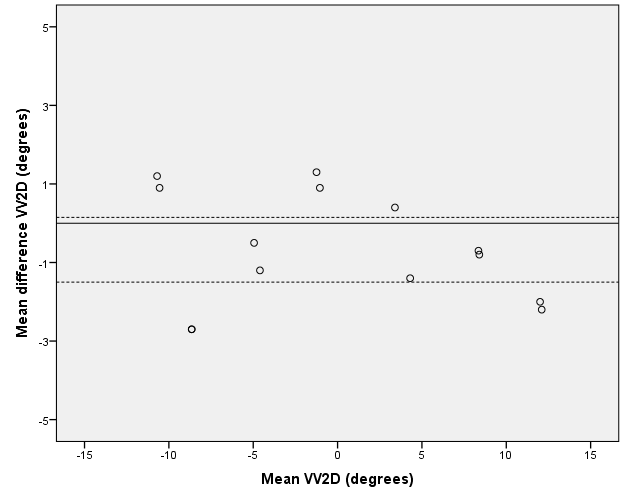

Supplement: S1 Fig — (TIF) [file pone.0146187.s002.tif]

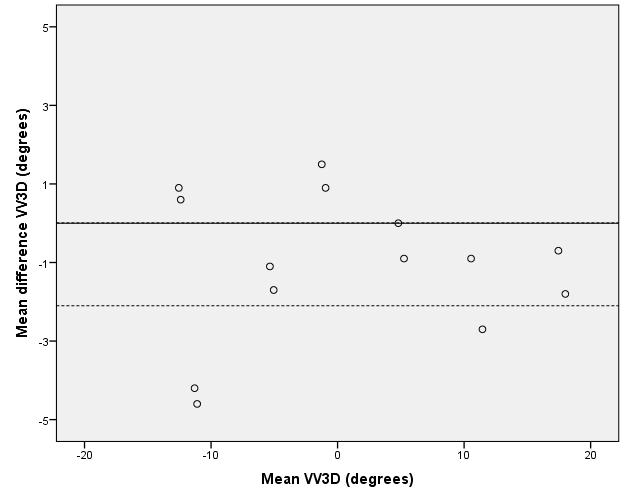

Supplement: S2 Fig — (TIF) [file pone.0146187.s003.tif]
